# Polymyxin B therapy based on therapeutic drug monitoring in carbapenem-resistant organisms sepsis: the PMB-CROS randomized clinical trial

**DOI:** 10.1186/s13054-023-04522-6

**Published:** 2023-06-13

**Authors:** Shaohua Liu, Ying Wu, Shaoyan Qi, Huanzhang Shao, Min Feng, Lihua Xing, Hongmei Liu, Yanqiu Gao, Zhiqiang Zhu, Shuguang Zhang, Yuming Du, Yibin Lu, Jing Yang, Pingyan Chen, Tongwen Sun

**Affiliations:** 1 Department of General ICU, The First Affiliated Hospital of Zhengzhou University, Henan Key Laboratory of Critical Care Medicine, Zhengzhou Key Laboratory of Sepsis, Henan Engineering Research Center for Critical Care Medicine, Zhengzhou, 450052 People’s Republic of China; 2 Department of Biostatistics, School of Public Health, Southern Medical University, Guangzhou, 510515 People’s Republic of China; 3 Department of ICU, The Second Affiliated Hospital of Zhengzhou University, Zhengzhou, 450052 People’s Republic of China; 4 Department of ICU, Henan Provincial People’s Hospital, Zhengzhou, 450052 People’s Republic of China; 5 Department of Surgery ICU, The First Affiliated Hospital of Zhengzhou University, Zhengzhou, 450052 People’s Republic of China; 6 Department of Respiratory ICU, The First Affiliated Hospital of Zhengzhou University, Zhengzhou, 450052 People’s Republic of China; 7 Department of Respiratory ICU, Henan Provincial People’s Hospital, Zhengzhou, 450052 People’s Republic of China; 8 Department of Respiratory ICU, Zhengzhou Central Hospital, Zhengzhou, 450052 People’s Republic of China; 9 Department of Emergency ICU, The First Affiliated Hospital of Zhengzhou University, Zhengzhou, 450052 People’s Republic of China; 10 Department of ICU, Xinyang Central Hospital, Xinyang, 464000 People’s Republic of China; 11 Department of Pharmacy, The First Affiliated Hospital of Zhengzhou University, Zhengzhou, 450052 People’s Republic of China

**Keywords:** Polymyxin B, Therapeutic drug monitoring, Carbapenem-resistant gram-negative bacteria, Severe infection, Optimization dose

## Abstract

**Background:**

The appropriate administration regimen of polymyxin B is yet controversial. The present study aimed to explore the optimal dose of polymyxin B under therapeutic drug monitoring (TDM) guidance.

**Methods:**

In China’s Henan province, 26 hospitals participated in a randomized controlled trial. We included patients with sepsis caused by carbapenem-resistant Gram-negative bacteria (CR-GNB) susceptible to polymyxin B. The patients were randomly divided into a high-dose (HD) group or a low-dose (LD) group and received 150 mg loading dose, 75 mg every 12 h and 100 mg loading dose, 50 mg every 12 h, respectively. TDM was employed to determine if the dose of polymyxin B needs adjustment based on the area under the concentration–time curve across 24 h at a steady state (ssAUC_0–24_) of 50–100 mg h/L. The primary outcome was the 14-day clinical response, and the secondary outcomes included 28- and 14-day mortality.

**Results:**

This trial included 311 patients, with 152 assigned to the HD group and 159 assigned to the LD group. Intention-to-treat analysis showed that the 14-day clinical response was non-significant (*p* = 0.527): 95/152 (62.5%) in the HD group and 95/159 (59.7%) in the LD group. Kaplan–Meier’s 180-day survival curve showed survival advantage in the HD group than in the LD group (*p* = 0.037). More patients achieved the target ssAUC_0–24_ in the HD than in the LD group (63.8% vs. 38.9%; *p* = 0.005) and in the septic shock subgroup compared to all subjects (HD group: 71.4% vs. 63.8%, *p* = 0.037; LD group: 58.3% vs. 38.9%, *p* = 0.0005). Also, the target AUC compliance was not correlated with clinical outcomes but with acute kidney injury (AKI) (*p* = 0.019). Adverse events did not differ between the HD and LD groups.

**Conclusion:**

A fixed polymyxin B loading dose of 150 mg and a maintenance dose of 75 mg every 12 h was safe for patients with sepsis caused by CR-GNB and improves long-term survival. The increased AUC was associated with increased incidence of AKI, and TDM results were valued to prevent AKI.

*Trial registration* Trial registration ClinicalTrials.gov: ChiCTR2100043208, Registration date: January 26, 2021.

**Supplementary Information:**

The online version contains supplementary material available at 10.1186/s13054-023-04522-6.

## Background

The increasing prevalence of multi-drug-resistant bacteria causes high attributable mortality, which is partially due to a lack of effective therapeutic drugs [1, 2]. Carbapenem-resistant Gram-negative bacteria (CR-GNB), also known as carbapenem-resistant organisms (CRO), including carbapenem-resistant Enterobacteriaceae (CRE), carbapenem-resistant *Acinetobacter baumannii* (CRAB), and carbapenem-resistant *Pseudomonas aeruginosa* (CRPA), are the main contributors of infectious diseases caused by multi-drug-resistant bacteria [3]. Although several new β-lactam antibiotics and β-lactamase inhibitors against CR-GNB with fewer side effects have been identified, many of them are not sold in China. In this event, polymyxin B, an old drug approved in the late 1950s and abandoned in the 1970s due to its toxicity, has been reintroduced to treat CR-GNB-caused infections [4, 5]. Polymyxins have a narrow therapeutic window and cause significant nephrotoxicity, which hinders their clinical application. Clinical guidelines recommend that the dosage of polymyxin B should be based on total body weight (TBW). A loading dose of 2.0–2.5 mg/kg (equivalent to 20,000–25,000 IU/kg) and a maintenance dose of 1.25–1.5 mg/kg (equivalent to 12,500–15,000 IU/kg) every 12 h may be appropriate, which is expected to achieve an area under the concentration–time curve across 24 h at a steady state (ssAUC_0–24_) target of 50–100 mg h/L (corresponding to average steady-state plasma concentration [C_ss, avg_] of 2–4 mg/L) [6]. Nevertheless, the most appropriate administration regimen for polymyxin B is controversial [7], and the therapeutic drug monitoring (TDM) of polymyxin B has not been widely used in clinics. Currently, there is little evidence that reaching a target therapeutic window of 50–100 mg h/L improves efficacy. In addition, weight-based strategies are being challenged by studies on the population pharmacokinetics (PK) of polymyxin B [8, 9].

Polymyxin B is the most commonly used polymyxin in China. However, the clinical regimen is yet to be standardized. Some studies have shown that the dosage of polymyxin B is lower than recommended in the guidelines and that doctors prefer to use a fixed daily dose of 50 mg or 75 mg every 12 h, which might not be according to TBW but it can be implemented easily and avoids wastage. Additionally, some patients did not receive the polymyxin B loading dose [10, 11]. To the best of our knowledge, prolonged exposure to low-concentration antibiotics may lead to bacterial resistance, and without employing the appropriate loading dose of polymyxin B, optimal plasma exposure cannot be achieved quickly [12], leading to treatment failure.

In this study, we conducted a randomized trial to explore the appropriate clinical administration regimen of polymyxin B for the treatment of severe infections caused by CR-GNB. Also, TDM was used to explain the polymyxin B dose–response correlation and determine the optimal dosage.

## Methods

### Study design and patients

This is an open, multicenter, randomized, controlled study conducted according to the principles of Helsinki Declaration and was approved by the ethics committees of the participating hospitals. Written informed consent was obtained from the patients before they were randomly assigned to various groups. When patients and had no capabilities due to consciousness disorders, sedative states, intense weakness, their families signed the informed consent, and the patient was required to sign a new informed consent if he/she regained capabilities during the trial.

A total of 26 hospitals in Henan province of China and all patients admitted from January 2021–2022 participated in this study. The inclusion criteria were as follows: (1) age 18–75 years; (2) suffered from severe infections caused by CR-GNB susceptible to polymyxin B (minimal inhibitory concentration [MIC] ≤ 2 mg/L by microdilution broth method) [13]; (3) clinical diagnosis of sepsis or septic shock as defined by sepsis 3.0 [14]; (4) infections included bacteremia, pneumonia, intraabdominal infection, skin and soft tissue infection, and central nervous system infection. This study excluded patients who had received polymyxin B treatment previously, pregnant or lactating women, patients with known polymyxin B allergies, and those enrolled in other trials.

### Randomization and masking

All patients meeting the inclusion criteria were divided into a high initial dose group or a low initial dose group according to age and infection sites in a randomized block design; the randomization was computer-generated, and the block size was set at 6. If a subject was considered eligible for enrollment, we queried the random information corresponding to the group number: the letter “A” for a high initial dose and the letter “B” for a low initial dose. Thus, the subjects were randomized equally into two groups. Participants and doctors were not blinded to the randomization, and the primary outcome was decided by two researchers who were unaware of the treatment arm.

### Procedures

The time from the diagnosis of CR-GNB infection to entering the trial and receiving polymyxin B treatment should not exceed 48 h. In the high-dose (HD) group, patients received 150 mg of polymyxin B (Shanghai First Biochemical Pharmaceutical Co., Ltd.) intravenously as a loading dose and 75 mg every 12 h as an initial maintenance dose, while in the low-dose (LD) group, patients received 100 mg of polymyxin B intravenously as an initial loading dose and 50 mg every 12 h as an initial maintenance dose. For TDM, blood samples were withdrawn from all dosages during the second and seventh dosages. Two blood samples were collected immediately before the infusion (2 mL, C_0h_) and 2 h (2 mL, C_2h_) after the beginning of the infusion for measurements.

Before analysis, the supernatant collected from blood samples was stored at − 80 °C. The plasma concentrations of polymyxin B, B1 and B2, were determined using a validated ultra-performance liquid chromatography-tandem mass spectrometry in our hospital laboratory, and the plasma concentration of polymyxin B was the sum of the above two polypeptides [15].

The limited sampling strategies of AUC_0–24_ were investigated using Bayesian and linear regression analyses based on previously published population PK model using Phoenix® NLME software (v8.3, Pharsight, Mountain View, CA, USA). The polymyxin B AUC_0–24_ was calculated using the following equation: AUC_0–24_ = 2 × (− 0.673 + 6.084 × C0h + 6.230 × C2h) [16]. Using this method, we determined the ssAUC_0–24_ of polymyxin B that reached a steady-state plasma level after the seventh dosage (on day 4).

If the ssAUC_0–24_ reached the target of 50–100 mg·h/L, we maintained the current dosage unchanged, but if not, the maintenance dose was increased or decreased by 25 mg every time on the day or the next day and plasma concentration of polymyxin B was rechecked  after four dose administration, until the daily dose of polymyxin B exceeded the range of 50–200 mg or the ssAUC_0-24_ reached the 50–100 mg·h/L window, new blood samples were collected. The dose of polymyxin B does not need to be adjusted according to renal function [12, 17, 18]. Patients were administered polymyxin B for at least 7 days or until either discharge or death. Except for polymyxin B, doctors could choose one or two anti-infective agents for combination therapy (i.e., β-lactam, aminoglycosides, cephalosporin, quinolone, oxazolidinone, minocycline, fosfomycin), according to bacterial drug resistance. Patients with clinical signs of infection were sampled every 72 h until two consecutive negative results were obtained.

### Outcomes

The primary outcome of this analysis was the 14-day clinical response rate, which was defined based on survival [19]: hemodynamic stability in patients with shock (mean blood pressure > 65 mmHg without vasopressor support), the improved or stable ratio of arterial partial pressure of oxygen/fraction of inspired oxygen (PaO_2_/FiO_2_) for patients with pneumonia, microbiological cure for patients with bacteremia (no growth in the blood of index isolate on day 14), and improved or stable Sequential Organ Failure Assessment (SOFA) score. (Baseline SOFA score ≥ 3 was improved by at least 30%, and for baseline SOFA < 3 the score remained the same or decreased.) Patients who fit the above description were assessed for clinical responses. The secondary outcomes included 28-day mortality, 14-day mortality, bacterial clearance rate, ventilated-free days at 28 days, length of stay in the intensive care unit, total in-hospital stay, superinfections, and adverse events.

### Statistical analysis

The primary analysis was based on intention-to-treat. Patients who survived for > 72 h after randomization were included in the per-protocol analysis. Considering the impact of population characteristics on outcomes, we conducted subgroup analysis in populations with septic shock or with different infection sites and bacteria.

Statistical analysis was conducted using SAS 9.4. The continuous variable data were subjected to Kolmogorov–Smirnov test to determine whether they adhered to normal distribution. Mean value (SD) or quartile (upper quartile Q1, median Q2, lower quartile Q3) was used to describe the baseline characteristics of continuous variables, and the percentage was used for descriptive analysis of counting variables.

The primary endpoints of patients lost to follow-up were deemed ineffective. A log-rank test was used to compare time-to-event endpoints, and patients without an event were censored up to the date last known to be at risk; also, Kaplan–Meier estimation was carried out. A chi-square test was used to examine the binary endpoints. For the risk difference and relative risk, Wald and Newcombe confidence intervals were offered, respectively. Continuous endpoints were analyzed with a two-independent sample t test or the Wilcoxon rank test. All the reported *p* values were two-sided.

Based on the primary outcomes, we calculated a sample size of 311 that could achieve 80% power to detect a relative risk of 1.260 at a significance level of 0.05, and the 14-day clinical response rate in the low-dose group was assumed to be 58.0%.

## Results

### Patients characteristics

**Fig. 1:**
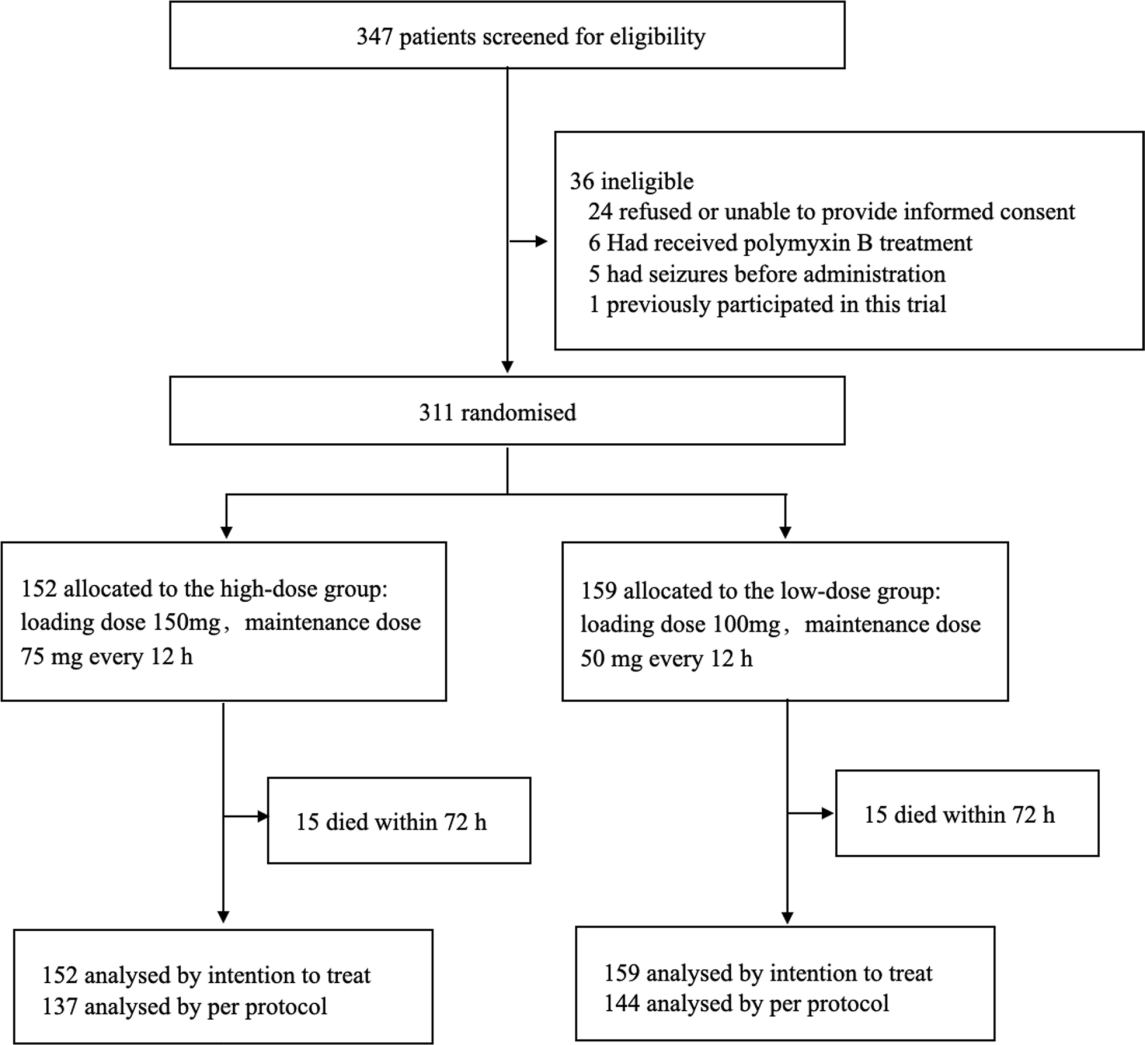
Trial profile

A total of 347 patients were screened between January 2021 and 2022, and of them, 36 were excluded. Finally, the trial comprised 311 patients, of whom 152 were randomly assigned to the HD group and 159 to the LD group (Fig. 1). The majority of patients (74%, 230/311) were males, 113 out of 311 (36.3%) patients had recently undergone trauma or surgery, and 241 out of 311 (77.5%) patients presented one or more comorbidities. The most common infection sites were lung (244/311, 78.5%) and blood (68/311, 21.9%), and the most common pathogens were CRAB (162/311; 52.1%) and CRE (145/311; 46.6%). Almost all the patients (300/311; 96.5%) received double and triple combinations of anti-infection therapy based on polymyxin B, of which carbapenems were the most common combination agents (167/311, 53.7%). The total 28-day mortality was 32.2% (107/311), and the total 180-day mortality was 75.3% (232/308).

Polymyxin B was administered at a lower dose in 159 patients than in 152 patients who received a higher dose. All of them received a loading dose; the HD group received a loading dose of 2.14 (interquartile range [IQR] 2, 2.5) mg/kg and a maintenance dose of 2.14 (IQR 2, 2.5) mg/kg/day, and the LD group received a loading dose of 1.54 (IQR 1.41, 1.75) mg/kg and a maintenance dose of 1.54 (IQR 1.41, 1.75) mg/kg/day. The median age of both groups was 58, and the average acute physiology and chronic health assessment (APACHE II) score were 18.7 in the HD group and 18.4 in the LD group. TBWs ranged from 45 to 99 kg (68.5 [IQR 60, 75]) in 150 patients in the HD group, and 2 patients were extremely obese at 120 kg and 180 kg, respectively. On the other hand, TBWs ranged from 40 to 100 kg (65.0 [IQR 57.0, 71.0]) in 159 patients in the LD group. The two groups were similar in most baseline characteristics and severity but not in TBW (70.0 [IQR 60.0, 75.0] vs. 65.0 [IQR 57.0, 71.0]; *p* = 0.006), which might be because gender was not considered during randomization (31 out of 152 [20.4%] in the HD group vs. 50 out of 159 [31.4%] in the LD group; *p* = 0.026]. This led to the inclusion of more female patients in the LD group, and the low weight of female patients resulted in the lower weight of patients in the LD group (Table 1).

**Table 1:** Patient and infection characteristics

Demographics and background	HD group (n = 152)	LD group (n = 159)	*p* value
Age, years, Q2 (Q1, Q3)	58 (48, 66)	58 (50, 68)	0.360
Gender			
Male, %	121 (79.6)	109 (68.6)	0.026
Female, %	31 (20.4)	50 (31.4)	
BMI, kg/m^2^, Q2 (Q1, Q3)	23.6 (21.6, 25.4)	22.9 (20.9, 24.5)	0.010
Weight, kg, Q2 (Q1, Q3)	70.0 (60.0, 75.0)	65.0 (57.0, 71.0)	0.006
Comorbidity, %			
Chronic heart disease	68 (44.7)	69 (43.4)	0.812
Chronic lung disease	14 (9.2)	15 (9.4)	0.946
Central nervous system diseases	67 (44.1)	55 (34.6)	0.087
Chronic kidney disease	7 (4.6)	5 (3.1)	0.504
Chronic liver disease	9 (5.9)	4 (2.5)	0.134
Diabetes	31 (20.4)	36 (22.6)	0.630
Solid malignancy	6 (3.9)	10 (6.3)	0.350
Hematological malignancy	0 (0.0)	2 (1.3)	0.499
Immune suppressive therapy	15 (9.9)	8 (5.0)	0.103
Other	9 (5.9)	7 (4.4)	0.545
Recent trauma or surgery	54 (35.5)	59 (37.1)	0.772
Infection site, %			
Pulmonary infection	122 (80.3)	122 (76.7)	0.449
Bloodstream infection	31 (20.4)	37 (23.3)	0.540
Abdominal infection	13 (8.6)	14 (8.8)	0.937
Intracranial infection	6 (3.9)	12 (7.5)	0.174
Skin and soft tissue infection	4 (2.6)	9 (5.7)	0.182
Other	9 (5.9)	6 (3.8)	0.377
Single-site infection	123 (80.9)	119 (74.8)	0.197
Two-site infection	21 (13.8)	34 (21.4)	0.080
Multi-site infection	8 (5.3)	6 (3.8)	0.527
Pathogen, %			
CRE	66 (43.4)	79 (49.7)	0.268
CRAB	80 (52.6)	82 (51.6)	0.852
CRPA	20 (13.2)	19 (11.9)	0.748
Other	2 (1.3)	3 (1.9)	1.000
Single-bacterial infection	135 (88.8)	137 (86.2)	0.480
Multi-bacterial infection	17 (11.2)	22 (13.8)	0.480
Mechanical ventilation (invasive), %	108 (71.1)	114 (71.7)	0.721
Hemodialysis, %	20 (13.2)	19 (11.9)	0.677
Temperature, °C, Q2 (Q1, Q3)	37.3 (36.8, 38.0)	37.4 (36.8, 38.2)	0.513
Mean artery pressure, mm Hg, M ± SD	86.8 ± 15.5	85.8 ± 15.4	0.579
Hemodynamic support, %	25 (16.4)	28 (17.6)	0.785
PaO_2_/FiO_2_, M ± SD	227.7 ± 94.9	233.4 ± 93.5	0.601
Scr, mg/dL, Q2 (Q1, Q3)	59.4 (40.0, 99.8)	62.5 (44.5, 97.9)	0.323
Albumin, g/dL, Q2 (Q1, Q3)	31.3 (28.0, 33.9)	31.3 (27.5, 37.0)	0.461
C-reactive protein, mg/L, Q2 (Q1, Q3)	75.2 (41.9, 141.0)	85.2 (38.0, 161.5)	0.399
D-dimer, Q2 (Q1, Q3)	3.7 (1.4,6.3)	2.8 (1.3, 5.9)	0.456
Lactic acid, mmol/L, Q2 (Q1, Q3)	1.4 (1.0,2.0)	1.6 (1.2, 2.1)	0.169
White blood cells, 10^9^/L, Q2 (Q1, Q3)	11.3 (8.0, 15.7)	11.4 (8.1, 15.1)	0.856
Platelet, 10^9^/L, Q2 (Q1, Q3)	168.5 (85.5, 289.8)	151.0 (79.0, 244.0)	0.244
Anti-infection therapy, %			
Polymyxin B monotherapy	6 (3.9)	5 (3.1)	0.702
Polymyxin B + carbapenem	44 (28.9)	47 (29.6)	0.906
Polymyxin B + tigecycline	13 (8.6)	18 (11.3)	0.415
Polymyxin B + β-lactam	27 (17.8)	24 (15.1)	0.525
Polymyxin B + ceftazidime avibactam	2 (1.3)	7 (4.4)	0.174
Polymyxin B + X	5 (3.3)	6 (3.8)	0.817
Polymyxin B + carbapenem + tegacyclin	21 (13.8)	25 (15.7)	0.636
Polymyxin B + tegacyclin + X	14 (9.2)	10 (6.3)	0.335
Polymyxin B + β-lactam + X	3 (2.0)	3 (1.9)	1.000
Polymyxin B + carbapenem + X	17 (11.2)	13 (8.2)	0.369
GCS, Q2 (Q1, Q3)	9.0 (6.0, 13.8)	8.0 (5.0, 14.0)	0.771
APACHE II score, M ± SD	19.0 ± 7.6	18.8 ± 6.9	0.819
SOFA score, Q2 (Q1, Q3)	7.0 (5.0, 10.0)	8.0 (5.0, 10.0)	0.774

Data are mean (SD), n (%), or median (IQR). HD: high dose. LD: low dose. BMI = body mass index. CRE = carbapenem-resistant Enterobacteriaceae. CRAB = carbapenem-resistant Acinetobacter baumannii. CRPA = carbapenem-resistant Pseudomonas aeruginosa. Scr: serum creatinine. GCS = Glasgow Coma Scale. APACHE = Acute Physiology and Chronic Health Evaluation. SOFA = Sequential Organ Failure Assessment. X = β-lactam / aminoglycosides / cephalosporin / quinolone / oxazolidinone / minocycline / fosfomycin

### Intention-to-treat analysis

No significant difference was observed in the 14-day clinical response between the HD and LD groups in the intention-to-treat analysis. 95/152 (62.5%) patients in the HD group and 95/159 (59.7%) patients in the LD group met the criteria for clinical response (risk difference [RD], 2.75%; relative risk [RR]: 1.046; 95% confidence interval [CI]: 0.876–1.249; *p* = 0.619) (Table 2).

**Table 2:** Outcomes for intention-to-treat population

Outcomes	HD group (*n* = 152)	LD group (*n* = 159)	RR or HR (95% CI)	RD (95% CI)	*p* value
14-day response, %	62.5 (95/152)	59.7 (95/159)	1.046 (0.876, 1.249)	2.75 (− 8.08, 13.58)	0.619
*Mortality*					
14-day mortality, %	23.7 (36/152)	27.8 (44/159)	0.856 (0.585, 1.252)	− 3.99 (− 13.57, 5.74)	0.421
28-day mortality,%	30.9 (47/152)	37.7 (60/159)	0.819 (0.601, 1.118)	− 6.81 (− 17.12, 3.73)	0.206
72-h mortality, %	9.9 (15/152)	9.4 (15/159)	1.046 (0.530, 2.066)	0.43 (− 6.13, 7.00)	0.897
*Disposition at day 28*					
Dead, %	30.9 (47/152)	37.7 (60/159)	0.819 (0.601,1.118)	− 6.81 (− 17.12, 3.73)	0.250
Alive, still in the ICU, %	25 (38/152)	30.2 (48/159)	0.828 (0.576,1.190)	− 5.19 (− 15.10,4.73)	0.306
Alive, discharged home, %	16.4 (25/152)	9.4 (15/159)	1.743 (0.956,3.175)	7.01 (− 0.42,14.45)	0.065
Alive, palliative care, %	2.6 (4/152)	1.9 (3/159)	1.395 (0.163,3.151)	0.74 (− 2.56,4.05)	0.718
Alive, still hospitalized, %	25 (38/152)	21.4 (34/159)	0.872 (0.317,1.721)	3.62 (− 5.76,13.00)	0.506
Bacterial clearance*					
Microbiological cure, %	35.2 (50/142)	33.3 (48/144)	1.056 (0.766, 1.456)	1.88 (− 9.03, 12.74)	0.738
Bacterial persistence, %	56.3 (80/142)	55.6 (80/144)	1.014 (0.826, 1.246)	0.78 (− 10.59, 12.13)	0.894
Superinfection, %	11.3 (16/142)	12.5 (18/144)	0.901 (0.479, 1.696)	− 1.23 (− 8.89, 6.44)	0.748
*Time-to-event data*					
VFD^#^, Q_2_ (Q_1_, Q_3_)	14 (6, 21)	15 (6, 22)	1.064 (0.831, 1.362)	Not Applicable	0.613
ICU-days^†^, Q_2_ (Q_1_, Q_3_)	21 (15, 31)	21 (13, 30)	0.924 (0.738, 1.158)	Not Applicable	0.495
Hospital-days^‡^, Q_2_ (Q_1_, Q_3_)	30 (19, 42)	28 (17, 45)	0.943 (0.752, 1.182)	Not Applicable	0.611

Data are n (%) or median (IQR). n: sample size. HD: high dose. LD: low dose. RR: relative risk. HR: hazard ratio. RD: risk difference. *: Superinfection and original Bacterial persistence co-exist in 6 patients, so the sum of the parts of bacterial clearance is greater than 100% #: Ventilated-free days at 28 days. †: Length of ICU stay. ‡: Length of hospital stay

**Fig. 2:**
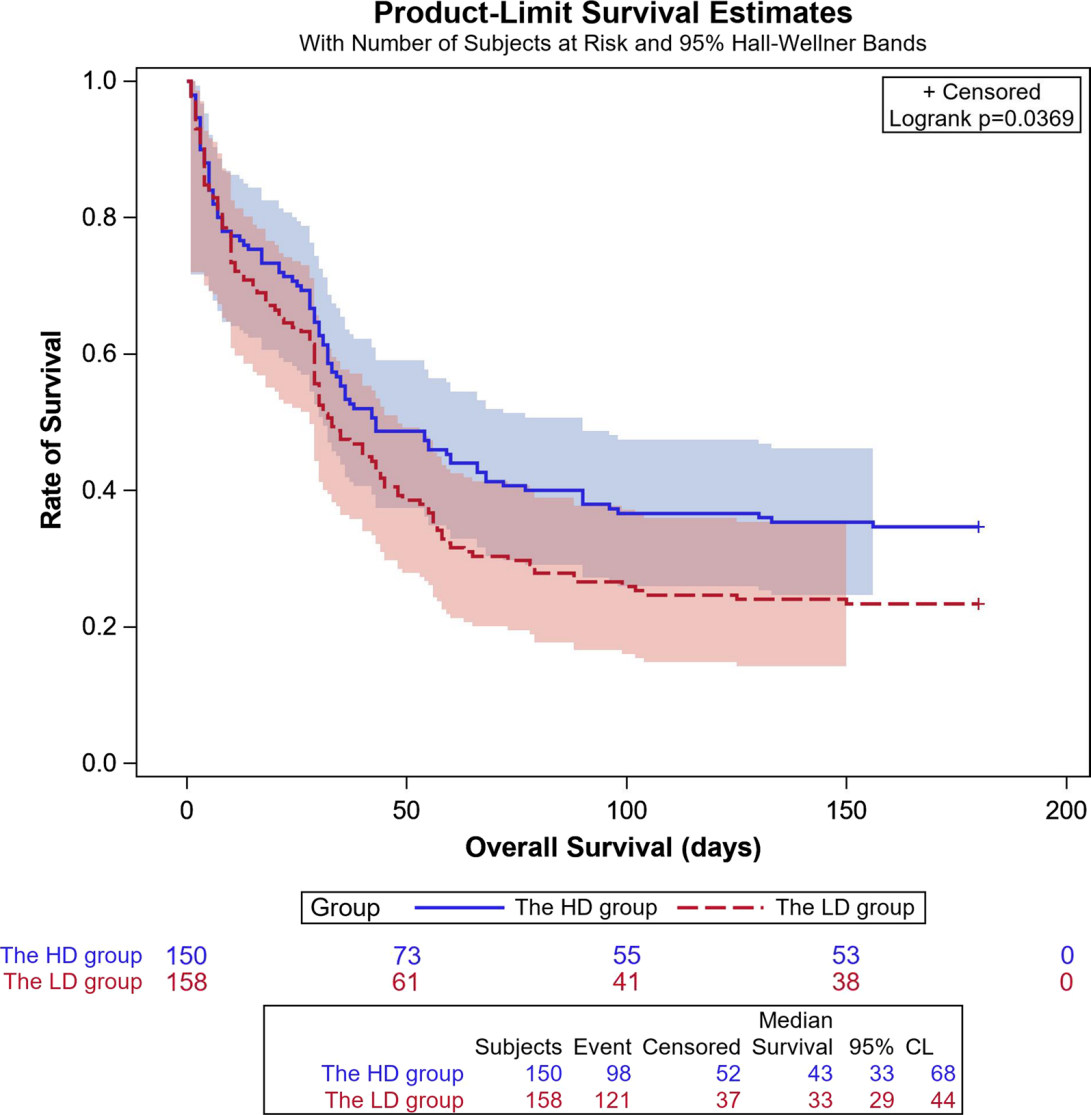
Kaplan–Meier’s survival curve of 180 days

Also, no significant difference was detected in all-cause mortality at 14 or 28 days after randomization. The 14-day mortality rate of the HD and LD groups was 23.7% (36/152) and 27.7% (44/159), respectively, and the RD was − 3.99% (95% CI − 13.57% to 5.74%). The 28-day mortality rate was 30.9% (47/152) and 37.7% (60/159) in the HD and LD groups, respectively (RD: − 6.8%, 95% CI − 17.12% to 3.73%). Subsequently, 25 (16.4%) patients in the HD group and 15 (9.4%) patients in the LD group were discharged from the hospital on day 28 after randomization (Table 2). A prolonged follow-up of 180 days reported 34.7% (52/150) survival rate in the HD group and 23.4% (37/158) in the LD group, and there was an obvious survival advantage in patients who received a high dose of polymyxin B; the Kaplan–Meier’s survival curve of 180 days showed that the hazard ratio (HR) was 0.754 (95% CI 0.577–0.984; *p* = 0.037) (Fig. 2).

### TDM

**Fig. 3:**
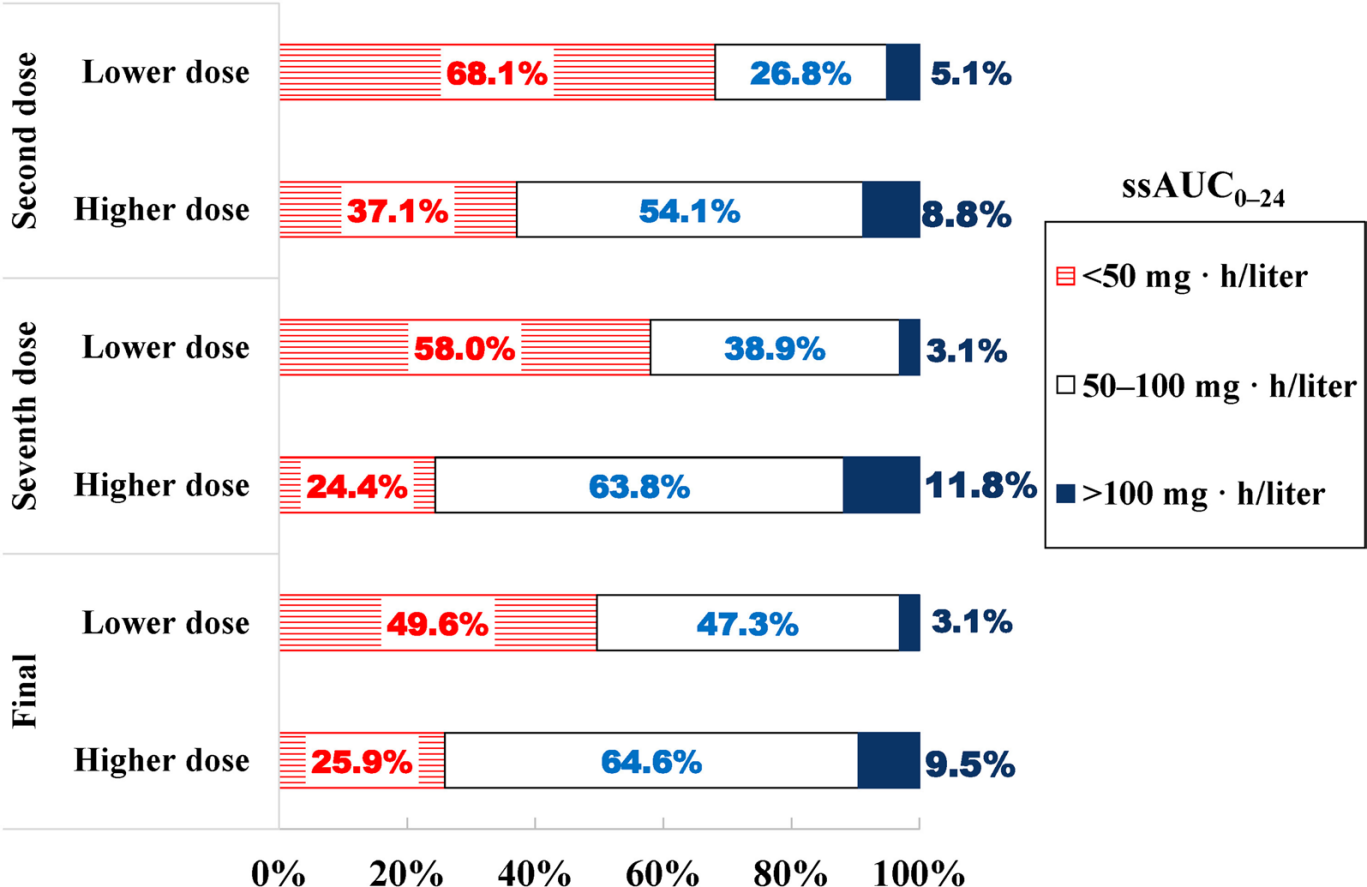
The target AUC compliance of polymyxin B

The ssAUC_0–24_ achieving rate (50–100 mg·h/L) of the HD group was significantly higher than that of LD group (63.8%, 81/127 vs. 38.9%, 51/131; RR, 1.638; 95% CI 1.274–2.106; *p* < 0.001) after the seventh dosage. Moreover, more patients exceeded 100 mg·h/L in the HD group than in the LD group (11.8, 15/127 vs. 5.1, 8/157; RR, 1.724; 95% CI 0.736–4.040; *p* = 0.007). Patients who did not hit the ssAUC_0-24_ target had their dosage adjusted, and finally, the ssAUC_0–24_ achieving a rate of 64.6% (82/127) in the HD group was significantly different from 47.3% (62/131) in the LD group (RR, 1.364; 95% CI 1.093–1.703, *p* = 0.005) (Table 3 and Fig. 3).

**Table 3:** The target AUC compliance of polymyxin B

	HD group n, %	LD group n, %	RR (95% CI)	RD (95% CI)	*p* value
ssAUC_0–24_ between 50–100 mg · h/L at the second dose, %	54.1 (80/148)	26.8 (42/157)	2.021 (1.499, 2.723)	27.30 (16.37, 37.35)	< 0.001
ssAUC_0–24_ > 100 mg · h/L at the second dose, %	8.8 (13/148)	5.1 (8/157)	1.724 (0.736, 4.040)	3.69 (− 2.17, 9.88)	0.204
ssAUC_0–24_ between 50 and100 mg · h/L at the seventh dose, %	63.8 (81/127)	38.9 (51/131)	1.638 (1.274, 2.106)	24.85 (12.68, 35.99)	< 0.001
ssAUC_0–24_ > 100 mg · h/L at the seven dose, %	11.8 (15/127)	3.1 (4/131)	3.868 (1.319, 11.340)	8.76 (2.35, 15.77)	0.007
ssAUC_0–24_ between 50–100 mg · h/L at final, %	64.6 (82/127)	47.3 (62/131)	1.364 (1.093, 1.703)	17.24 (5.12, 28.65)	0.005
ssAUC_0–24_ > 100 mg · h/L at final, %	9.5 (12/127)	3.1 (4/131)	3.095 (1.025, 9.343)	6.40 (0.37, 13.00)	0.033

n: sample size. HD: high dose. LD: low dose. RR: risk ratio. RD: risk difference

Among the 258 patients who reported the ssAUC_0–24_ after the seventh dosage, 150 required dose adjustments due to failure to reach the target AUC. However, the plasma concentration of polymyxin B was rechecked in only 48 (32%) patients, while in the remaining 102 patients, we were unable to adjust the dosage or recheck for the progression of the disease, death, transfer out of the ICU, or discharge from the hospital. Subsequently, 18/48 (37.5%) patients did not reach the target AUC after the first dose adjustment. Among these, 7 patients underwent a second dose adjustment.

### Subgroup analysis

**Fig. 4:**
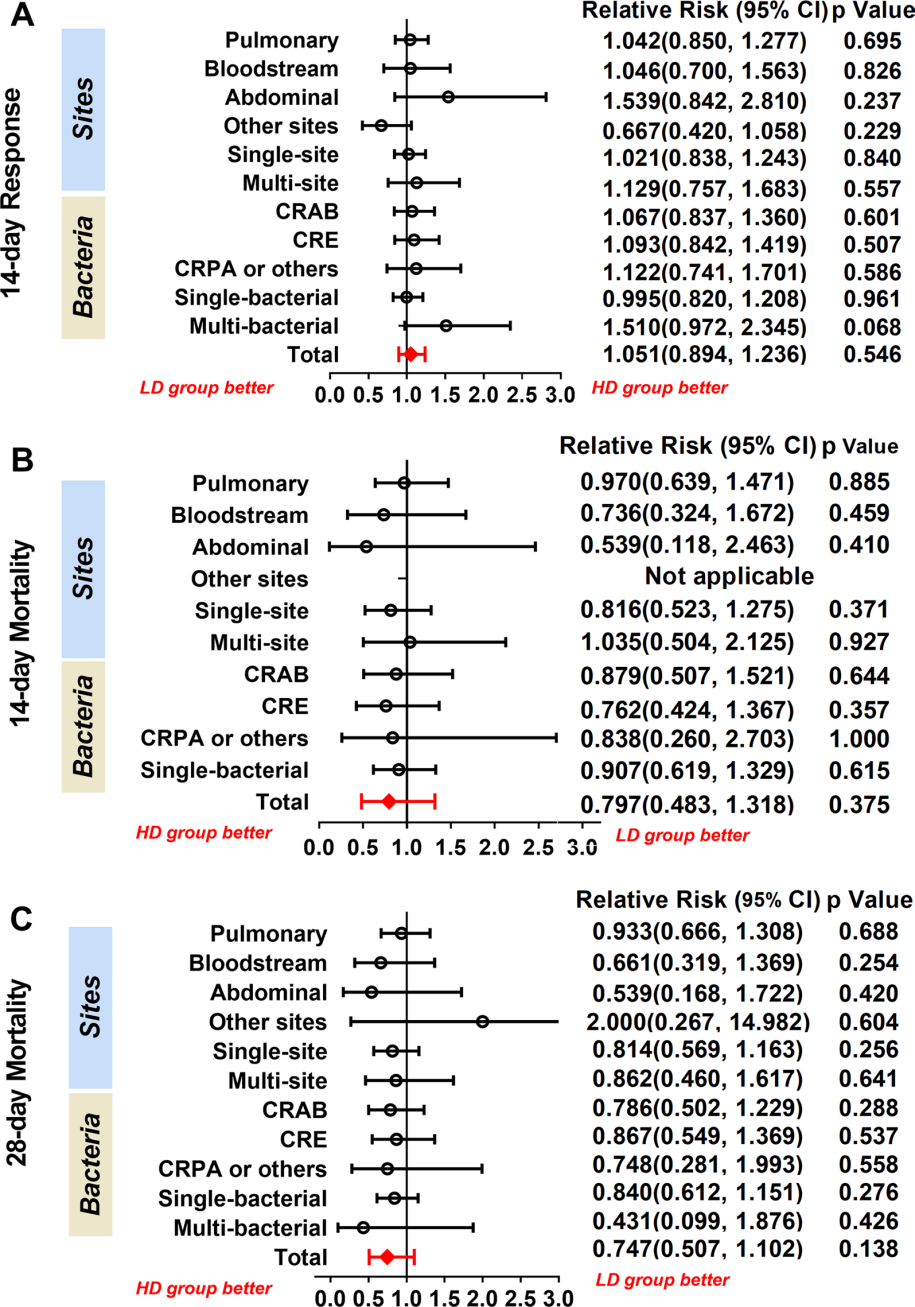
Forest plots for subgroup analysis of infection sites and bacteria. CRE = carbapenem-resistant Enterobacteriaceae. CRAB = carbapenem-resistant Acinetobacter baumannii. CRPA = carbapenem-resistant Pseudomonas aeruginosa. Results for subgroup infected at other sites and subgroup infected with multiple bacteria are not applicable since a denominator value of zero is illegal

A total of 137 participants in the HD group and 144 participants in the LD group who survived for more than 72 h constituted the per-protocol population. The results are similar to those obtained in the intention-to-treat analysis; no difference was observed in 14-day clinical response, 14-day mortality, and 28-day mortality (Additional file 1: Table S1). Also, the infection sites and pathogens were not related to clinical outcomes (Fig. 4 and Additional file 1: Table S2).

Subgroup analysis of participants with septic shock showed that the primary and secondary outcomes did not differ markedly (Table 4). 15/21 (71.4%) patients in the HD septic shock subgroup and 14/24 (58.3%) patients in the LD septic shock subgroup reached the ssAUC_0-24_ standard, which was significantly higher than that in all subjects (HD group: 71.4% vs. 63.8%, RR: 1.11; 95% CI 0.593–1.627, *p* = 0.037; LD: 58.3% vs. 38.9%, RR: 1.498; 95% CI 0.810–2.186, *p* = 0.0005).

**Table 4:** Subgroup analysis results for patients with septic shock

Outcomes	HD group (*n* = 25)	LD group (*n* = 28)	RR or HR (95% CI)	RD (95% CI)	*p* value
14-day response, %	48.0 (12/25)	50.0 (14/28)	0.960 (0.553, 1.666)	− 2.0 (− 27.0, 23.4)	0.884
*Mortality*					
14-day mortality, %	24.0 (6/25)	32.1 (9/28)	0.747 (0.309, 1.802)	− 8.1 (− 30.5, 15.9)	0.511
28-day mortality, %	40.0 (10/25)	53.6 (15/28)	0.747 (0.413, 1.348)	− 13.6 (− 37.3, 12.6)	0.323
Bacterial Clearance*					
Microbiological cure, %	34.8 (8/23)	32.0 (8/25)	1.087 (0.489, 2.419)	− 2.8 (22.5, − 27.9)	0.838
Bacterial persistence, %	65.2 (15/23)	52.0 (13/25)	1.254 (0.776, 2.028)	13.2 (− 13.9, 37.7)	0.354
Superinfection, %	8.7 (2/23)	16.0 (4/25)	0.544 (0.110, 2.692)	− 7.3 (− 27.0, 13.2)	0.445
*Time-to-event data*					
VFD^#^, Q_2_(Q_1_, Q_3_)	10 (0, 22)	14 (8, 18)	0.947 (0.536, 1.674)	Not Applicable	0.798
ICU-days^†^, Q_2_(Q_1_, Q_3_)	23 (15, 35)	22 (12, 26)	0.726 (0.416, 1.267)	Not Applicable	0.271
Hospital-days^‡^, Q_2_(Q_1_, Q_3_)	28 (20, 39)	28 (16, 46)	1.112 (0.640, 1.930)	Not Applicable	0.725
OS, Q_2_(Q_1_, Q_3_)	31 (17, 60)	25 (10, 48)	0.768 (0.434, 1.359)	Not Applicable	0.369
*ssAUC*_*0–24*_* at the seventh dose*					
50–100 mg · h/L, %	71.4 (15/21)	58.3 (14/24)	1.225 (0.794, 1.888)	13.1 (− 14.4, 37.6)	0.360
> 100 mg · h/L, %	14.3 (3/21)	0.0 (0/24)	Not Applicable	14.3 (− 2.4, 34.6)	0.055
*Adverse events*					
AE, %	52.0 (13/25)	50.0 (14/28)	1.04 (0.613, 1.764)	2.0 (− 23.4, 27.0)	0.884
AKI, %	16.0 (4/25)	21.4 (6/28)	0.747 (0.238, 2.345)	− 5.4 (− 25.9, 16.3)	0.614
KIDGO Stage 1	50.0 (2/4)	50.0 (3/6)	1.000 (0.282, 3.544)	0.0 (46.9, 46.9)	1.000
KIDGO Stage 2	25.0 (1/4)	33.3 (2/6)	0.750 (0.098, 5.768)	− 8.3 (− 50.3, 42.5)	1.000
KIDGO Stage 3	25.0 (1/4)	16.7 (1/6)	1.500 (0.127, 17.667)	8.3 (−36.3, 55.3)	1.000

Data are n (%) or median (IQR). n: sample size. HD: high dose. LD: low dose. RR: relative risk. HR: hazard ratio. RD: Risk Difference. *: Superinfection and original Bacterial persistence co-exist in 2 patients, so the sum of the parts of bacterial clearance is greater than 100% ^#^: Ventilated-free days at 28 days. ^†^: Length of ICU stay. ^‡^: Length of hospital stay. OS: overall survival. AE: adverse events. AKI: acute kidney injury. KDIGO: Kidney Disease Improving Global Outcomes

### Sensitivity analysis

Since convective solute removal used in hemofiltration (for example, CVVH) significantly reduces the plasma concentration of polymyxin B [20], statistical analysis on the patients without CRRT or without CVVH did not detect any significant difference (Additional file 1: Tables S3 and S4).

The target AUC compliance rate had no correlation with the clinical outcomes (Additional file 1: Table S5). No difference was detected in both primary and secondary outcomes, but the incidence of acute renal injury (AKI) varies significantly between the groups (*p* = 0.019); it was 14.0% (15/107) in patients with ssAUC_0-24_ < 50 mg ·h/L, 26.5% (35/132) with ssAUC_0-24_ in 50–100 mg ·h/L, and 36.8% (7/19) with ssAUC_0-24_ exceeding 100 mg ·h/L. We also attempted to analyze the mortality in patients with septic shock, and the results showed that the 28-day mortality was 61.5% in patients with an AUC < 50 mg ·h/L and 34.5% in patients with an AUC between 50 and 100 mg h/L, and there is no difference between them (*p* = 0.248) (Additional file 1: Table S6).

The comparison of baseline characteristics between sepsis patients with and without shock showed that the level of serum creatinine (Scr) and lactate was significantly higher in patients with septic shock compared to sepsis patients without shock (Additional file 1: Table S7). The mean level of Scr was 116.7 ± 87.1 mg/dL in septic shock patients and 86.3 ± 86.3 mg/ dL in sepsis patients without shock (*p* = 0.001). The mean level of lactate was 4.5 ± 15.6 mmol/L in septic shock patients and 2.3 ± 8.7 mmol/L in sepsis patients without shock (*p* = 0.001).

### Adverse events

The frequency of total adverse events and individual adverse events did not differ significantly between the HD and LD groups; the two groups reported 48.7 (74/152) and 43.4 (69/159) total adverse events, respectively (RR 0.891; 95% CI 0.70–1.134; *p* = 0.350). AKI is the most frequent adverse event, occurring in 30/152 (19.7%) and 32/159 (20.1%) of the HD and LD groups, respectively. According to the Kidney Disease Improving Global Outcomes (KDIGO) guidelines, the majority of patients had stage 1 [17/30 (56.7%) vs. 21/31 (67.7%)] and stage 2 [10/30 (33.3%) vs. 8/31 (25.8%)] AKI [21]. In addition, diarrhea [28/152 (18.4%) vs. 29/159 (18.2%)], pigmentation [24/152 (15.8%) vs. 20/159 (12.6%)], and superinfection [17/152 (11.2%) vs. 20/159 (12.6%)] were common adverse events in the two groups (Table 5), while no relevant adverse event was detected after discontinuation of treatment.

**Table 5:** The adverse events

	HD group (*n* = 152, %)	LD group (*n* = 159, %)	RR (95% CI)	*p* value
AE	48.7 (74/152)	43.4 (69/159)	0.891 (0.700,1.134)	0.350
AKI	19.7 (30/152)	20.1 (32/159)	1.020 (0.653,1.592)	0.932
KIDGO Stage 1	56.7 (17/30)	67.7 (21/31)	1.195 (0.804,1.777)	0.372
KIDGO Stage 2	33.3 (10/30)	25.8 (8/31)	0.774 (0.354,1.693)	0.519
KIDGO Stage 3	10.0 (3/30)	6.5 (2/31)	0.645 (0.116,3.593)	0.614
Diarrhea	18.4 (28/152)	18.2 (29/159)	0.999 (0.619,1.583)	0.967
Pigmentation	15.8 (24/152)	12.6 (20/159)	0.797 (0.460,1.381)	0.417
Superinfection	11.2 (17/152)	12.6 (20/159)	1.125 (0.613,2.064)	0.704
Coagulation abnormalities	6.6 (10/152)	5.7 (9/159)	0.860 (0.359,2.059)	0.735
Paresthesia	0.7 (1/152)	0.0 (0/159)	Not Applicable	0.489

Data are n (%). n: sample size. HD: high dose. LD: low dose. RR: risk ratio. AE: adverse events. AKI: acute kidney injury. KDIGO: Kidney Disease Improving Global Outcomes. The RR for paresthesia is not applicable since a numerator value of zero is illegal

## Discussion

The clinical and microbiological outcomes of different doses of polymyxin B in the treatment of sepsis caused by CR-GNB were similar, although more patients in the HD group (63.8%) achieved a ssAUC_0–24_ target of 50–100 mg·h/L after the seventh dose compared to the LD group (38.9%) (*p* < 0.001). Irrespective of high dose [loading dose: 150 mg (equivalent to 1,500,000 IU), maintenance dose: 75 mg every 12 h] or a low (loading dose: 100 mg, maintenance dose: 50 mg every 12 h) initial therapeutic dose, there is no significant difference in 14-day response of the patients. The 28-day mortality rate of the HD group (30.9%) was 6.8% lower than that of the LD group (37.7%), but the difference was not statistically significant. After prolonged follow-up, we found that the 180-day survival in the HD group was higher than that in the LD group. This result could be attributed to prolonged illness and increased long-term mortality due to low exposure dose of antibiotics. However, we also observed that the 180-day survival of both groups was extremely low. This phenomenon could be related to the critical condition and numerous complications of patients infected with CR-GNB. The most frequent adverse event is AKI, followed by superinfection, pigmentation, and diarrhea. The frequency of adverse events did not differ significantly between the two groups.

This is the first randomized controlled trial comparing different polymyxin B doses in the treatment of severe CR-GNB infections under TDM. Based on the results of a published murine thigh infection model study, when the MIC of polymyxin B to CR-GNB is ≤ 2 mg/L, the lower bound of the target window is estimated to be a ssAUC_0–24_ of 50 mg h/L, and the upper limit of the therapeutic window for polymyxin B is estimated to be a ssAUC_0–24_ of 100 mg ·h/L [9]. The current guidelines recommend a loading dose of 2.0–2.5 mg/kg (equivalent to 20,000–25,000 IU/kg) and a maintenance dose of 1.25–1.5 mg/kg (equivalent to 12,500–15,000 IU/kg) every 12 h for polymyxin B based on TBW [6]. However, no appropriate dosing regimen has been deduced for polymyxin B. According to the guidelines for the administration of a standard dose of polymyxin B, Monte Carlo simulations showed that only 71% of simulated subjects achieved a ssAUC_0–24_ target of 50–100 mg·h/L [9]. Thus, an appropriate initial dose and a rapid method of TDM would be required to improve the efficacy of polymyxin B.

In the current study, only a minority of patients (32%) who failed to achieve the target AUC received dosage adjustments and rechecked plasma concentration of polymyxin B. Such a finding indicated that TDM could not be carried out smoothly in clinical practice because it is time-consuming and requires multiple blood samples at different time points to determine whether ssAUC_0–24_ meets the standard value.​ Also, more than one-third of patients failed to achieve the target AUC after the first dose adjustment, suggesting that the method of dose adjustment with a 25 mg increase or decrease may be simple to use but insufficient to achieve the target AUC rapidly. Dose adjustment may be depends on the difference between the measured and target AUC, prompting us to explore suitable dosage adjustment strategies using the Bayesian approach in the future.

Interestingly, a higher proportion of patients reached the ssAUC_0-24_ standard of 50–100 mg ·h/L in the septic shock subgroup compared to the total group. Hence, we compared some baseline characteristics between sepsis patients with and without shock to explain why target AUC compliance rates were high in more severe patients, and the results showed that the level of Scr was significantly higher in patients with septic shock and the plasma concentration of polymyxin B appeared to increase with increasing Scr; a similar phenomenon was described in previous studies [22, 23].

Data from our prospective study indicated there was no correlation between whether ssAUC_0-24_ met the criteria and clinical outcomes; however, the 28-day mortality in septic shock patients with a ssAUC_0-24_ of polymyxin B between 50 and 100 mg ·h/L showed a decreasing trend, although not statistically significant due to limited sample size; also, AKI increased with increasing AUC, which is consistent with our previous findings in a real-world cohort of patients [24]. Septic shock is a severe subtype of sepsis with high mortality [25]. Briefly, critically ill patients may experience increased plasma concentrations of polymyxin B due to renal dysfunction, but the severity of the disease has a greater impact on outcomes than the benefit of increased AUC.

The most accurate PK/PD index for colistin is the ratio of the area under the unbound concentration–time curve to the MIC (fAUC/MIC), which is also applicable to polymyxin B. In murine thigh infection models, the fAUC/MIC value for 2-log bacterial killing was approximately 20 [26]. In order to reach a fAUC/MIC value of 20, the Monte Carlo simulations showed that a daily dose of 3 mg/kg/day should be considered for severe infections caused by CR-GNB with polymyxin B MIC of ≤ 2 mg/L [12]. The high doses of polymyxin B are frequently constrained in clinical practice due to nephrotoxicity. This study reported the incidence of AKI as 19.7% (30/152) and 20.1% (32/159) in the HD and LD groups, respectively, and more than half of them had mild renal toxicity (Class I of KDIGO classification). Such AKI incidence seems acceptable because severe infection can also lead to renal impairment.

Some studies speculated that the total dose of polymyxin B is highly related to both efficacy and toxicity, irrespective of patient weight [27]. In a retrospective cohort study, the polymyxin B dose of ≥ 200 mg/day (corresponding to 2.5–3 mg/kg/day in patients weighing 80–65 kg) was independently associated with low hospital mortality, although 119 (50.6%) presented some degree of renal impairment during therapy. The findings speculated that the survival benefits of high doses of drugs outweighed the risk of nephrotoxicity [28]. Another cohort study of 58 patients with sepsis who received a high-dose polymyxin B (median daily dose of 3.2 mg/kg/day) for ≥ 72 h showed promising mortality rates; among them, 25 (58.1%) patients developed AKI [29]. Therefore, regimens containing > 3 mg/kg/day of polymyxin B should not be recommended due to a lack of clinical data on safety. On the other hand, reducing the daily dosage of polymyxin B might weaken the efficacy of antibiotic therapy. A retrospective cohort study showed that polymyxin B dosages of < 1.3 mg/kg/day were associated with 30-day mortality in patients with renal impairment [30]. Xiao et al. collected data from 10,066 Gram-negative organisms isolated from patients with bloodstream infections (BSIs) to optimize the balance between efficacy and toxicity in different populations. Fixed and weight-based polymyxin B maintenance dose was simulated using Monte Carlo method. The results showed that the appropriate loading dose is 2.5 mg/kg of polymyxin B regardless of renal function, followed by a fixed maintenance dose of 60 mg every 12 h in patients with impaired renal function and 1.25 mg/kg every 12 h in patients with normal renal function [31]. However, these simulated data have not yet been substantiated.

Our previous real-world study of 100 patients with CR-GNB infections treated with polymyxin B found that the 28-day mortality was 40%, and about 50% of patients in the study were administered a fixed daily dose of 100 mg of polymyxin B [11]. Another retrospective study from China involving 268 similar patients claimed that the clinical efficacy rate was 36.57%, and the all-cause mortality rate was 33.96%, in this study, only 110/268 (41.04%) patients were administered a loading dose, and after calculation based on TBW, the median loading dose was 1.01 mg/kg and the median maintenance dose was 0.85 mg/kg [10]. In our current study, the total 28-day mortality was 32.2% (107/311), with 30.9% (47/152) in the HD group and 37.7% (60/159) in the LD group. Compared to the above study, our regimens of polymyxin B showed promise for the 28-day mortality, especially in patients who received a loading dose of 150 mg and a maintenance dose of 75 mg every 12 h. This phenomenon could be partially attributed to the fact that every patient in our study received a loading dose of polymyxin B, which helped in achieving optimal plasma exposure at the earliest [12].

Nevertheless, the present trial has several limitations, which might explain the lack of clinical difference between the two groups treated with different doses of polymyxin B for severe CR-GNB infection. First, there was sample shortage and selection bias. We may be able to make some inferences if the sample size is increased because a 7% difference was detected in the 28-day mortality between the two groups. Moreover, patients who received high doses of polymyxin B had elevated body weight which could have affected the results. Next, the dosage of polymyxin B in both groups was lower than the standard recommended dosage; the higher initial dose group received a mean maintenance dose of 2.14 (IQR 2, 2.5) mg/kg/day, and the lower initial dose group received a mean maintenance dose of 1.54 (IQR 1.41, 1.75) mg/kg/day, and only 64.6% and 47.3% achieved ssAUC_0–24_ values within the target therapeutic window of 50–100 mg·h/L. Due to unscientific usage, the therapeutic effects may not be satisfactory in the two groups. Finally, the recommended PK/PD exposure targets should be applied to polymyxin B monotherapy. However, in the current study, almost all patients received combination therapy with one or two anti-infective agents in addition to polymyxin B, which might affect the final clinical outcomes. We did not limit the combination therapy with polymyxin B because this is still a mainstream treatment pattern in China. A recent study showed that combination therapy with colistin and meropenem was not superior to colistin monotherapy for the treatment of pneumonia or BSI caused by drug-resistant (XDR) *Acinetobacter baumannii*, XDR *Pseudomonas aeruginosa*, and CRE [32]. Additional studies are warranted to fully understand the role of polymyxin B combination therapy.

Patients with CR-GNB infections have various diseases and poor physical condition, which are likely to affect clinical outcomes. Accumulating evidence showed that polymyxin B has differential efficacy according to types [33] and sites [26] of CR-GNB infections. Thus, in the future, randomized controlled trials will be required for various patient subgroups and anti-infection strategies.

## Conclusions

For patients with sepsis caused by CR-GNB, a fixed polymyxin B loading dose of 150 mg followed by a maintenance dose of 75 mg every 12 h was safe, with better 180-day survival compared to patients receiving a lower dose. The target AUC compliance of polymyxin B was not correlated with clinical outcomes, but increased AUC was associated with increased incidence of AKI. Therefore, the TDM should be valued to prevent AKI in sepsis patients.

## Supplementary Information

**Additional file 1**: **Table S1.** Outcomes for per protocolpopulation. **Table S2.** Subgroup analyses of infection site and bacteria. **Table S3.** Subgroup analysis results for patients without CRRT. **Table S4.** Subgroup analysis results for patients without CVVH. **Table S5.** Association between the target AUC compliance and clinical outcomes. **Table S6.** Association between the target AUC compliance and clinical outcomes in septic shock patients. **Table S7.** Baseline characteristics for sepsis patients with and without shock. 

## Data Availability

The data supporting the findings of the article are available on request by contacting the corresponding author.
