# Supplementary material for: Polymyxin B therapy based on therapeutic drug monitoring in carbapenem-resistant organisms sepsis: the PMB-CROS randomized clinical trial
Source: Crit Care. 2023 Jun 13;27:232. doi: 10.1186/s13054-023-04522-6 (PMC10262552; doi:10.1186/s13054-023-04522-6)
Supplement: Supplementary file 1 — Additional file 1: Table S1. Outcomes for per protocolpopulation. Table S2. Subgroup analyses of infection site and bacteria. Table S3. Subgroup analysis results for patients without CRRT. Table S4. Subgroup analysis results for patients without CVVH. Table S5. Association between the target AUC compliance and clinical outcomes. Table S6. Association between the target AUC compliance and clinical outcomes in septic shock patients. Table S7. Baseline characteristics for sepsis patients with and without shock. [file 13054_2023_4522_MOESM1_ESM.docx]

**Additional file 1. Additional results.**

*Table S1-*Outcomes for per protocol population.

|  | HD group  n=137, % | LD group  *n=144*, % | RR (95% CI) | RD (95% CI)  % | p *val*ue |
| --- | --- | --- | --- | --- | --- |
| 14-day response | 69.3%(95/137) | 66.0%(95/144) | 1.051(0.894, 1.236) | 3.37(-7.53, 14.12) | 0.546 |
| 14-day mortality | 16.1%(22/137) | 20.1%(29/144) | 0.797(0.483, 1.318) | -4.08(-13.04, 5.01) | 0.375 |
| 28-day mortality | 23.4%(32/137) | 31.3%(45/144) | 0.747(0.507, 1.102) | -7.89(-18.05, 2.55) | 0.138 |

Data are n (%). n: sample size. HD: high-dose. LD: low-dose. RR: risk ratio. RD: risk difference.

*Table S2-*Subgroup analyses of infection site and bacteria.

|  | | HD group  *n*, % | LD group  *n*, % | RR (95% CI) | RD (95% CI), % | *p v*alue |
| --- | --- | --- | --- | --- | --- | --- |
| Infection site |  | | | | | |
| 14-day response rate | | | | | | |
| Pulmonary | | 61.5(75/122) | 59.0(72/122) | 1.042(0.850, 1.277) | 2.46(-9.70, 14.51) | 0.695 |
| Bloodstream | | 59.4(19/32) | 56.8(21/37) | 1.046(0.700, 1.563) | 2.62(-19.86, 24.51) | 0.826 |
| Abdominal | | 76.9(10/13) | 50.0(7/14) | 1.539(0.842, 2.810) | 26.92(-8.81, 54.49) | 0.237 |
| Other | | 66.7(6/9) | 100.0(6/6) | 0.667(0.420, 1.058) | -33.33(-64.58, 11.12) | 0.229 |
| Single-site infection | | 62.6(77/123) | 61.3(73/119) | 1.021(0.838, 1.243) | 1.26(-10.82, 13.31) | 0.840 |
| Multi-site infection | | 62.1(18/29) | 55.0(22/40) | 1.129(0.757, 1.683) | 7.07(-15.97, 28.57) | 0.557 |
| 14-day mortality | | | | | | |
| Pulmonary | | 26.2(32/122) | 27.1(33/122) | 0.970(0.639, 1.471) | -0.82(-11.82, 10.21) | 0.885 |
| Bloodstream | | 21.9(7/32) | 29.7(11/37) | 0.736(0.324, 1.672) | -7.85(-27.23, 13.00) | 0.459 |
| Abdominal | | 15.4(2/13) | 28.6(4/14) | 0.539(0.118, 2.463) | -13.19(-41.51, 18.51) | 0.410 |
| Other | | 33.3(3/9) | 0.0(0/6) | Not applicable | 33.33(-11.12, 64.58) | 0.229 |
| Single-site infection | | 22.0(27/123) | 26.9(32/119) | 0.816(0.523, 1.275) | -4.94(-15.66, 5.87) | 0.371 |
| Multi-site infection | | 31.0(9/29) | 30.0(12/40) | 1.035(0.504, 2.125) | 1.03(-19.64, 22.79) | 0.927 |
| 28-day mortality | | | | | | |
| Pulmonary | | 34.4(42/122) | 36.9(45/122) | 0.933(0.666, 1.308) | -2.46(-14.27, 9.45) | 0.688 |
| Bloodstream | | 25.0(8/32) | 37.8(14/37) | 0.661(0.319, 1.369) | -12.84(-32.74, 9.12) | 0.254 |
| Abdominal | | 23.1(3/13) | 42.9(6/14) | 0.539(0.168, 1.722) | -19.78(-48.50, 14.86) | 0.420 |
| Other | | 33.3(3/9) | 16.7(1/6) | 2.000(0.267, 14.982) | 16.67(-28.36, 50.77) | 0.604 |
| Single-site infection | | 30.1(37/123) | 37.0(44/119) | 0.814(0.569, 1.163) | -6.89(-18.51, 4.95) | 0.256 |
| Multi-site infection | | 34.5(10/29) | 40.0(16/40) | 0.862(0.460, 1.617) | -5.52(-26.70, 17.21) | 0.641 |
| Bacteria |  | | | | | |
| 14-day response rate | | | | | | |
| CRAB | | 63.8(51/80) | 59.8(49/82) | 1.067(0.837, 1.360) | 3.99(-10.79, 18.51) | 0.601 |
| CRE | | 63.6(42/66) | 58.2(46/79) | 1.093(0.842, 1.419) | 5.41(-10.41, 20.66) | 0.507 |
| CRPA or others | | 71.4(15/21) | 63.6(14/22) | 1.122(0.741, 1.701) | 7.79(-19.30, 33.20) | 0.586 |
| Single-bacterial infection | | 60.0(81/135) | 60.3(82/136) | 0.995(0.820, 1.208) | -0.29(-11.80, 11.22) | 0.961 |
| Multi-bacterial infection | | 82.4(14/17) | 54.6(12/22) | 1.510(0.972, 2.345) | 27.81(-2.03, 50.76) | 0.068 |
| 14-day mortality | | | | | | |
| CRAB | | 22.5(18/80) | 25.6(21/82) | 0.879(0.507, 1.521) | -3.11(-16.08, 10.05) | 0.644 |
| CRE | | 21.2(14/66) | 27.9(22/79) | 0.762(0.424, 1.367) | -6.64(-20.10, 7.61) | 0.357 |
| CRPA or others | | 19.1(4/21) | 22.7(5/22) | 0.838(0.260, 2.703) | -3.68(-27.31, 20.77) | 1.000 |
| Single-bacterial infection | | 26.7(36/135) | 29.4(40/136) | 0.907(0.619, 1.329) | -2.75(-13.31, 7.91) | 0.615 |
| Multi-bacterial infection | | 0.0(0/17) | 18.2(4/22) | Not applicable | -18.18(-38.52, 3.22) | 0.118 |
| 28-day mortality | | | | | | |
| CRAB | | 28.8(23/80) | 36.6(30/82) | 0.786(0.502, 1.229) | -7.84(-21.75, 6.55) | 0.288 |
| CRE | | 31.8(21/66) | 36.7(29/79) | 0.867(0.549, 1.369) | -4.89(-19.75, 10.57) | 0.537 |
| CRPA or others | | 23.8(5/21) | 31.8(7/22) | 0.748(0.281, 1.993) | -8.01(-32.69, 18.29) | 0.558 |
| Single-bacterial infection | | 33.3(45/135) | 39.7(54/136) | 0.840(0.612, 1.151) | -6.37(-17.56, 5.05) | 0.276 |
| Multi-bacterial infection | | 11.8(2/17) | 27.3(6/22) | 0.431(0.099, 1.876) | -15.51(-38.04, 11.12) | 0.426 |

Data are n (%) or median (IQR). n: sample size. HD: high-dose. LD: low-dose. RR=risk ratio. RD=risk difference CRE= carbapenem-resistant Enterobacteriaceae. CRAB=carbapenem-resistant Acinetobacter baumannii. CRPA= carbapenem-resistant Pseudomonas aeruginosa. Results for subgroup infected at other sites in 14-day mortality and subgroup infected with multiple bacteria are not applicable since a denominator value of zero is illegal.

*Table S3-* Subgroup analysis results for patients without CRRT.

| Outcomes | HD group  (n=125, %) | LD dose group  (n=137, %) | RR or HR  (95% CI) | Risk Difference (95% CI) | *p* value |
| --- | --- | --- | --- | --- | --- |
| 14-day response | 63.2(79/125) | 64.2(88/137) | 0.984(0.819, 1.182) | -1.0(-12.6, 10.5) | 0.862 |
| 14-day mortality | 23.2(29/125) | 23.4(32/137) | 0.993(0.640, 1.543) | -0.2(-10.3, 10.1) | 0.976 |
| 28-day mortality | 29.6(37/125) | 33.6(46/137) | 0.882(0.616, 1.262) | -4.0(-15.0, 7.3) | 0.490 |
| Bacterial Clearance* | | | | | |
| Microbiological failure | 34.8(41/118) | 36.0(45/125) | 0.965(0.687, 1.356) | -1.3(-13.1, 10.7) | 0.838 |
| Bacterial persistence | 55.9(66/118) | 53.6(67/125) | 1.044(0.830, 1.312) | 2.3(-10.1, 14.6) | 0.715 |
| Superinfection | 11.0(13/118) | 12.0(15/125) | 0.918(0.457, 1.847) | -1.0(-9.2, 7.3) | 0.810 |
| Time-to-event data | | | | | |
| VFD^#^, Q_2_(Q_1_, Q_3_) | 15(7, 22) | 15(6, 22) | 1.073(0.819, 1.405) | Not Applicable | 0.611 |
| ICU-days^†^, Q_2_(Q_1_, Q_3_) | 20(14, 28) | 21(14, 32) | 1.009(0.791, 1.288) | Not Applicable | 0.936 |
| Hospital-days^‡^, Q_2_(Q_1_, Q_3_) | 30(20, 43) | 29(19, 46) | 0.982(0.769, 1.255) | Not Applicable | 0.879 |
| OS, Q_2_(Q_1_, Q_3_) | 43(17, -) | 37(15, 125) | 0.767(0.573, 1.027) | Not Applicable | 0.075 |
| ssAUC_0–24_ at the seventh dose | | | | | |
| 50–100 mg h/L | 63.8(67/105) | 38.6(44/114) | 1.653(1.259, 2.172) | 25.2(12.0, 37.2) | <0.001 |
| >100 mg h/L | 12.4(13/105) | 2.6(3/114) | 4.705(1.379,16.049) | 9.8(2.8, 17.6) | 0.006 |
| Adverse events | | | | | |
| AE | 50.4(63/125) | 43.8(60/137) | 1.151(0.890, 1.489) | 6.6(-5.4, 18.4) | 0.285 |
| AKI | 22.4(28/125) | 20.4(28/137) | 1.096(0.689, 1.744) | 2.0(-7.9, 12.0) | 0.700 |
| KIDGO Stage 1 | 60.7(17/28) | 63.0(17/27) | 0.964(0.637, 1.461) | -2.3(-26.2, 22.2) | 0.864 |
| KIDGO Stage 2 | 32.1(9/28) | 29.6(8/27) | 1.085(0.491, 2.400) | 2.5(-21.1, 25.6) | 0.840 |
| KIDGO Stage 3 | 7.1(2/28) | 7.4(2/27) | 0.964(0.146, 6.366) | -0.3(-17.0, 16.1) | 0.970 |

Data are n (%) or median (IQR). n: sample size. HD: high-dose. LD: low-dose. RR: relative risk. HR: hazard ratio. *: Superinfection and original Bacterial persistence co-exist in 6 patients, so the sum of the bacterial clearance fractions was greater than 100%. ^#^: Ventilated-free days at 28 days. ^†^: Length of ICU stay. ^‡^: Length of hospital stay. OS: overall survival.

*Table S4-* Subgroup analysis results for patients without CVVH.

| Outcomes | HD group  (n=142, %) | LD group  (n=152, %) | RR or HR  (95% CI) | Risk Difference (95% CI) | *p* value |
| --- | --- | --- | --- | --- | --- |
| 14-day response | 63.4(90/142) | 61.8(94/152) | 1.025(0.859, 1.223) | 1.5(-9.5, 12.4) | 0.785 |
| 14-day mortality | 23.2(33/142) | 25.7(39/152) | 0.906(0.605, 1.356) | -2.4(-12.1, 7.4) | 0.630 |
| 28-day mortality | 30.3(43/142) | 35.5(54/152) | 0.852(0.614, 1.184) | -5.2(-15.8, 5.5) | 0.339 |
| Bacterial Clearance* | | | | | |
| Microbiological cure | 37.0(50/135) | 34.3(48/140) | 1.080(0.786, 1.484) | 2.8(-8.5, 13.9) | 0.634 |
| Bacterial persistence | 54.8(74/135) | 55.0(77/140) | 0.997(0.804, 1.235) | -0.2(-11.8, 11.4) | 0.975 |
| Superinfection | 11.1(15/135) | 12.1(17/140) | 0.915(0.476, 1.758) | -1.0(-8.8, 6.8) | 0.790 |
| Time-to-event data | | | | | |
| VFD^#^, Q_2_(Q_1_, Q_3_) | 14(6, 21) | 14(6, 22) | 1.086(0.843, 1.397) | Not Applicable | 0.515 |
| ICU-days^†^, Q_2_(Q_1_, Q_3_) | 20(14, 30) | 21(14, 30) | 0.962(0.764, 1.211) | Not Applicable | 0.742 |
| Hospital-days^‡^, Q_2_(Q_1_, Q_3_) | 30(19, 43) | 29(18, 45) | 0.966(0.766, 1.217) | Not Applicable | 0.760 |
| OS, Q_2_(Q_1_, Q_3_) | 43(17, -) | 35(10, 115) | 0.776(0.591, 1.019) | Not Applicable | 0.068 |
| ssAUC_0–24_ at the seventh dose | | | | | |
| 50–100 mg h/L | 65.0(78/120) | 40.8(51/125) | 1.593(1.242, 2.043) | 24.2(11.7, 35.6) | <0.001 |
| >100 mg h/L r | 12.5(15/120) | 3.2(4/125) | 3.906(1.334,11.435) | 9.3(2.6, 16.7) | 0.007 |
| Adverse events | | | | | |
| AE | 50.0(71/142) | 44.1(67/152) | 1.134(0.890, 1.447) | 5.9(-5.4, 17.1) | 0.309 |
| AKI | 21.1(30/142) | 20.4(31/152) | 1.036(0.663, 1.619) | 0.7(-8.5, 10.1) | 0.877 |
| KIDGO Stage 1 | 56.7(17/30) | 66.7(20/30) | 0.850(0.568, 1.271) | -10.0(-32.5, 14.0) | 0.426 |
| KIDGO Stage 2 | 33.3(10/30) | 26.7(8/30) | 1.250(0.573, 2.727) | 6.7(-16.0, 28.5) | 0.573 |
| KIDGO Stage 3 | 10.0(3/30) | 6.7(2/30) | 1.500(0.270, 8.345) | 3.3(-12.7, 19.7) | 0.640 |

Data are n (%) or median (IQR). n: sample size. HD: high-dose. LD: low-dose. RR: relative risk. HR: hazard ratio. *: Superinfection and original Bacterial persistence co-exist in 6 patients, so the sum of the bacterial clearance fractions was greater than 100%. ^#^: Ventilated-free days at 28 days. ^†^: Length of ICU stay. ^‡^: Length of hospital stay. OS: overall survival. AE: adverse events. AKI: acute kidney injury. KDIGO: Kidney Disease Improving Global Outcomes.

*Table S5* -Association between the target AUC compliance and clinical outcomes. Association between the target AUC compliance and clinical outcomes in septic shock patients. Baseline characteristics for sepsis patients with and without shock.

| Outcomes | ssAUC_0–24_ at the seventh dose | | | *p* value |
| --- | --- | --- | --- | --- |
|  | <50 mg · h/L  (n=107) | 50–100 mg · h/L  (n=132) | >100 mg · h/L  (n=19) |  |
| 14-day response, % | 72.0(77/107) | 66.7(88/132) | 57.9(11/19) | 0.412 |
| 14-day mortality, % | 17.8(19/107) | 18.2(24/132) | 15.8(3/19) | 0.968 |
| 28-day mortality, % | 27.1(29/107) | 30.0(39/132) | 21.1(4/19) | 0.721 |
| Bacterial Clearance* | | | | |
| Microbiological failure, % | 35.4(34/96) | 42.4(53/125) | 31.6(6/19) | 0.458 |
| Bacterial persistence, % | 50.0(48/96) | 50.4(63/125) | 52.6(10/19) | 0.978 |
| Superinfection, % | 17.7(17/96) | 8.8(11/125) | 21.1(4/19) | 0.091 |
| Time-to-event data | | | | |
| VFD^#^, Q_2_(Q_1_, Q_3_) | 12(5, 19) | 11(5, 19) | 14(5, 20) | 0.947 |
| ICU-days^†^, Q_2_(Q_1_, Q_3_) | 26(20, 37) | 24(17, 37) | 21(15, 31) | 0.158 |
| Hospital-days^‡^, Q_2_(Q_1_, Q_3_) | 42(29, 98) | 58(24, -) | 42(18, -) | 0.203 |
| OS, Q_2_(Q_1_, Q_3_) | 38(25, 49) | 34(23, 50) | 30(21, 42) | 0.651 |
| Adverse events | | | | |
| AE, % | 47.7(51/107) | 53.0(70/132) | 42.1(8/19) | 0.551 |
| AKI, % | 14.0(15/107) | 26.5(35/132) | 36.8(7/19) | 0.019 |
| KIDGO Stage 1 | 78.6(11/14) | 54.3(19/35) | 42.9(3/7) | 0.193 |
| KIDGO Stage 2 | 21.4(3/14) | 37.1(13/35) | 28.6(2/7) | 0.555 |
| dKIDGO Stage 3 | 0.0(0/14) | 8.6(3/35) | 28.6(2/7) | 0.095 |

Data are n (%) or median (IQR). n: sample size. RR: relative risk. HR: hazard ratio. *: Superinfection and original Bacterial persistence co-exist in 6 patients, so the sum of the parts of bacterial clearance is greater than 100% ^#^: Ventilated-free days at 28 days. ^†^: Length of ICU stay. ^‡^: Length of hospital stay. OS: overall survival. AE: adverse events. AKI: acute kidney injury. KDIGO: Kidney Disease Improving Global Outcomes.

*Table S6* -Association between the target AUC compliance and clinical outcomes in septic shock patients. Baseline characteristics for sepsis patients with and without shock.

| Outcomes | ssAUC_0–24_ at the seventh dose | | | *p* value |
| --- | --- | --- | --- | --- |
|  | <50 mg · h/liter  (n=13) | 50–100 mg · h/liter  (n=29) | >100 mg · h/liter  (n=3) |  |
| 14-day response, % | 46.2(6/13) | 58.6(17/29) | 33.3(1/3) | 0.582 |
| 14-day mortality, % | 30.8(4/13) | 13.8(4/29) | 33.3(1/3) | 0.387 |
| 28-day mortality, % | 61.5(8/13) | 34.5(10/29) | 33.3(1/3) | 0.248 |

Data are n (%). n: sample size.

*Table S7 –* Baseline characteristics for sepsis patients with and without shock.

| Baseline  characteristics | Septic shock  (*n*=53) | Sepsis without shock  (*n*=258) | *p* value |
| --- | --- | --- | --- |
| Age, years, mean(*std*) | 58.6(12.4) | 55.7(13.0) | 0.141 |
| Weight, kg, mean(*std*) | 65.1(13.7) | 67.8(12.6) | 0.169 |
| BMI, kg/m^2^, mean(*std*) | 22.8(3.7) | 23.6(3.9) | 0.178 |
| Scr, mg/dL mean(*std*) | 116.7(87.1) | 86.3(86.3) | 0.001**^¶^** |
| PaO2/FiO2, mean(*std*) | 224.5(90.7) | 231.8(94.9) | 0.614 |
| Albumin, g/dL, mean(*std*) | 31.5(6.6) | 31.9(5.6) | 0.641 |
| Lactate, mmol/L, mean(*std*) | 4.5(15.6) | 2.3(8.7) | 0.001**^¶^** |

**^¶^** *t* tests were performed on data after logarithmic transformation for a better normality. *n*: sample size. BMI: body mass index. Scr: serum creatinine, *Std*: standard deviation.
